# Post-stroke arrhythmia could be a potential predictor for post-stroke depression

**DOI:** 10.1038/s41598-024-59789-8

**Published:** 2024-04-20

**Authors:** Tao Xu, Fangying Dong, Muhua Zhang, Kewu Wang, Tian Xu, Shudong Xia, Chao Feng

**Affiliations:** 1 Department of Cardiology, The Fourth Affiliated Hospital, Zhejiang University School of Medicine, Shangcheng Avenue N1#, Yiwu, Zhejiang China; 2 The Second Hospital of Jiaxing, The Second Affiliated Hospital of Jiaxing University, Jiaxing, China

**Keywords:** Cardiology, Medical research, Neurology

## Abstract

Post-stroke depression (PSD) is regarded as the consequence of multiple contributors involving the process of cognition, mood and autonomic system, with the specific mechanism unclear yet. As a common type of stroke-heart syndromes, post-stroke arrhythmia shared some common pathogenesis with PSD. We presumed that post-stroke arrhythmia might be an early distinguishable marker for the presence of PSD and aimed to verity their association in this study. Patients with first-ever ischemic stroke were enrolled. The presence of post-stroke ectopic arrhythmia and the symptoms of arrhythmia were recorded with anti-arrhythmia drugs prescribed when necessary. Patients were followed up 3 months later to identify their presence and severity of PSD using Hamilton Depression Scale (HAMD) and also presence and severity of arrhythmia. Characteristics including the prevalence of various types of arrhythmias were compared between PSD and non-PSD groups. The HAMD scores were compared between patients with and without arrhythmia in PSD group. Logistic regression was used to identify the independent predictor of PSD. Patients with PSD had higher prevalence of post-stroke arrhythmia especially newly-detected arrhythmia, symptomatic arrhythmia and poor-controlled arrhythmia. In PSD group, patients of post-stroke arrhythmia had higher scores of HAMD than those without arrhythmia. Presence of newly-detected, symptomatic and poor-controlled arrhythmias were independent predictor of PSD. post-stroke arrhythmia especially newly-detected arrhythmia and symptomatic arrhythmia could be an early predictor of PSD. Successful control of arrhythmia was associated with reduced prevalence and severity of PSD.

## Introduction

Stroke is a common disease among elderly with the prevalence estimated to be about 1115/100,000 according to a recent survey in China^1^. Stroke is associated with a series of adverse outcome, including severe handicap and death^2,3^, not only because of the neurological deficits caused by stroke directly, but also because of various complications of stroke, such as post-stroke depression (PSD)^4,5^.

PSD is quite common after stroke with the prevalence estimated to be more than 30%^4,6,7^, and is associated with severe handicap, poor response to rehabilitation and elevated long-term mortality of stroke^8^. Considering the adverse effect of PSD on the prognosis of stroke, it’s quite valuable to identify some distinguishable features which could predict the occurrence of PSD and enable early diagnosis. Plenty of studies focused on the association between lesion location and PSD, however the results are quite controversial and make the prediction of PSD based on the characteristics of infarctions on brain images quite unreliable^9–11^. Now it seems to be a consensus that PSD is not just a result of stroke in some special locations, but is caused by dysfunction of multiple networks involving the process of cognition, mood, immune and autonomic nervous system^12,13^.

Autonomic nervous system has been proven to be involved in multiple physiological process including the regulation of heart rate. Dysregulation of autonomic nervous system is also one of the major mechanisms of arrhythmia including post-stroke arrhythmia, which is one of the major forms of post-stroke cardiac events or so-called “stroke-heart syndrome”^5,14^. Differently from PSD, post-stroke arrhythmia usually occurred early after stroke and could be diagnosed easily based on the results of electrocardiogram, mainly including various subtypes of ectopic beats/arrhythmia and atrial fibrillation^14,15^. Previously, most studies focused on the impact of stroke on the presence of cardiac events. Few of them investigated the impact of post-stroke cardiac events on the progress of stroke in return, such as the occurrence and treatment of PSD. Considering PSD and post-stroke arrhythmia shared some common pathogenesis, some types of arrhythmias such as atrial fibrillation have already been proven to be associated with the presence of depression among patients without stroke^16,17^, it’s reasonable to presume that post-stroke arrhythmia could be a distinguishable and early predictor for PSD, and might even be involved in the pathogenesis of PSD. In order to test this hypothesis, in this study, we investigated the heart rhythm of patients after stroke, and tried to analyze its association with the presence of PSD.

## Materials and methods

### Study setting and patient population

This was a cohort study based on the patients attending to the Fourth Affiliated Hospital of Zhejiang University. Patients were consecutively enrolled if they met the criteria including: (1) first-ever ischemic stroke identified by magnetic resonance (MR) scan including diffusive-weighted images; (2) having will and ability to give consent to this study and finish all the evaluations; (3) age 18–80 years old. Patients were excluded if they had any of the following conditions: (1) previous history of stroke, brain tumor, Parkinson’s disease, or other central nervous system diseases; (2) previous history of depression, anxiety or drug dependence; (3) moderate to severe cognitive dysfunction with a Mini-Mental State Examination (MMSE) score < 18; (4) severe aphasia or dysarthria which enabled effective communication; (5) current diagnosis of cancer, other severe or end-stage disease; (6) died during hospitalization. This study was reviewed and approved by the Institutional Ethical Committee of the Fourth Affiliated Hospital Zhejiang University School of Medicine. All methods were performed in accordance with the declaration of Helsinki, the relevant guidelines and regulations of the Institutional Ethical Committee, with written informed consent for participation obtained from every patient or authorized family members in accordance with the national legislation and the institutional requirements.

### In-hospital management and evaluation

After admission, Histories of hypertension, diabetes, coronary artery disease, ectopic arrhythmia, use of anti-arrhythmic drugs (AADs) before stroke, smoking and current drinking were recorded. Previous history of arrhythmia was defined as the presence of atrial fibrillation, atrial flutter, frequent premature contraction which counted for more than 1% of 24-h electrocardiogram and atrial/supraventricular/ventricular tachycardia which lasts for more than 30 beats. Bradycardia, atrial-ventricular block and fascicular block were excluded from the category of arrhythmia in this study. Patients’ body max index was calculated. Each patient underwent MR scan to identify the presence, locations (left and right hemisphere, infratentorial area) and subtypes (lacunar and non-lacunar infarction) of acute infarction. The severity of periventricular and deep white matter hyperintensities was recorded to be 0–3 using Fazekas scale based on brain MRI^18^. The subtypes of acute infarctions were identified based on the TOAST-classification (Large-artery atherosclerosis, cardioembolism, small-artery occlusion, other determined etiology and undetermined etiology). At the first day of hospitalization, the severity of neurological deficit and cognitive impairment after stroke was evaluated using the National Institutes of Health Stroke Scale (NIHSS) and Mini-mental State Examination (MMSE) respectively. The heart rhythm after stroke was recorded using portable cardiac monitor (Mindray BeneHeart A3) or 3-day ambulatory electrocardiogram and interpreted by experienced cardiologists using Baihui Electrocardiographic analysis platform. *Post-stroke arrhythmia* was defined as occurrence of ectopic arrhythmia including atrial fibrillation, atrial flutter, frequent premature contractions (> 1% of all heart beats) or atrial/ventricular/supraventricular tachycardia (more than 30 beats) regardless of previous history of arrhythmia. *Newly-detected arrhythmia* was identified the occurrence of above-mentioned arrhythmia without previous history or record of arrhythmia. The subtypes of above-mentioned arrhythmia and the presence of arrhythmia-associated symptoms after stroke such as palpitation were recorded for each patient. AAD was prescribed for part of patients after consultation of cardiologists and was adjusted during follow-up by cardiologists mainly based on patient’s heart rate, symptom and frequency of arrhythmia. Other management of stroke was based on the recommendation of Chinese guideline for the management of ischemic stroke mainly including antiplatelet or anticoagulation therapy, statins, control of blood pressure and blood glucose if necessary, and early rehabilitation^19^.

### Follow-up

Three months later, patients were interviewed face-to-face in outpatient clinic. The severity of arrhythmia was assessed again based on the patients’ report and result of 24-h ambulatory electrocardiogram by cardiologists. For patients who took AADs after stroke onset, the remission/disappearance of arrhythmia-associated symptoms or the decrease of ectopic premature contractions/tachycardia for at least 30% was identified to indicate well-response to AADs, otherwise the patients should be regarded to have AAD-irresponsive arrhythmia. The functional outcome was evaluated using Barthel Index (BI) and Modified Rankin Scale (mRS). The occurrence of PSD was evaluated using Hamilton Depression Scale (HAMD) and Diagnostic and Statistical manual of mental disorders, 4th edn (DSM-IV) by experienced psychologists. Specifically, patients were diagnosed as PSD if they presented symptoms described in the clinical criteria of DSM-IV and had a score ≥ 7 evaluated by the HAMD. According to the presence of PSD, patients were categorized into PSD group and non-PSD group.

### Statistical analysis

All data were analyzed with SPSS 21.0 (SPSS Inc.). First, categorical variables including sex, education level, histories of hypertension, diabetes, coronary artery disease, arrhythmia, smoking and drinking, stroke subtypes, prevalence of post-stroke arrhythmia, newly-detected arrhythmia, symptomatic arrhythmia, and AAD-irresponsive arrhythmia were listed as proportion (number) and were compared using chi-square test between patients with and without PSD. Continuous variables including age, the severity of periventricular/deep WMH, NIHSS score, MMSE score and Barthel Index were listed as mean ± standard deviation and were compared between two groups using Student’ t test or Kruskal–Wallis test depending on the normality of data tested by Kolmogorov–Smirnov test. Second, logistic regression models were constructed to identify the independent risk factors for the presence of PSD, with the above-mentioned variables added in the models. Considering the interference among variables about arrhythmia, prevalence of previous history of arrhythmia, post-stroke arrhythmia, newly-detected arrhythmia, symptomatic arrhythmia and AAD-irresponsive-arrhythmia were added to five different models respectively. Furthermore, in order to explore the impact of arrhythmia and corresponding treatment on the severity of PSD, the HAMD score was compared between patients with and without post-stroke arrhythmia, newly-detected arrhythmia, symptomatic arrhythmia and AAD-irresponsive-arrhythmia using Student’ t test among patients with PSD. A P value < 0.05 was defined to indicate statistical difference.

In this study “Events per variable” (EPV) method which is widely accepted as an effective and simple means for the estimation of sample size was used. Specifically, EPV was presumed to be 10, the rate of endpoint events (PSD) was estimated to be around 1/3, and the number of covariates of logistic regression models were 17, therefore, the estimated sample size should be around 510.

### Informed consent

Informed consent is obtained from all participants.

## Results

**Figure 1:**
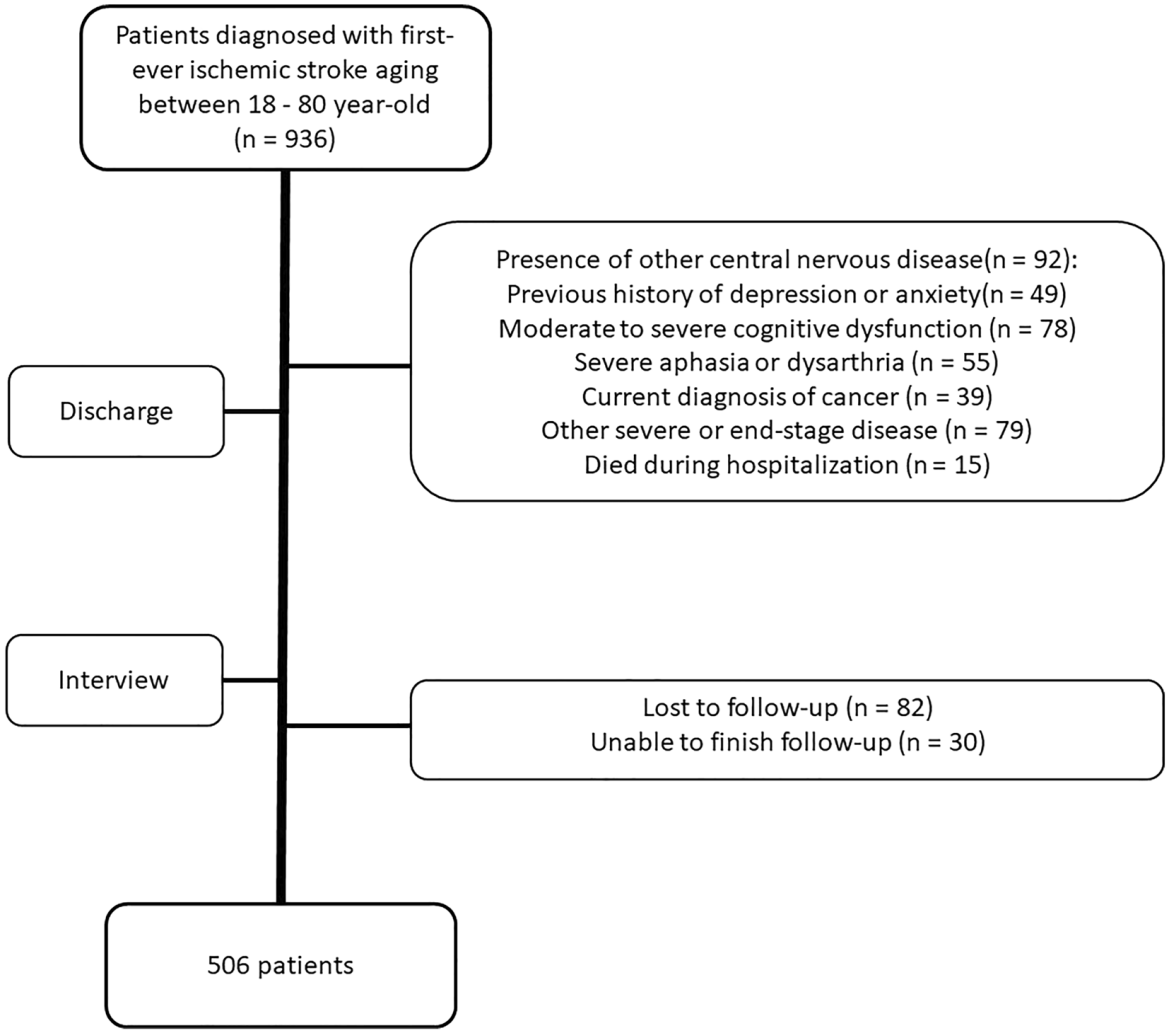
The flowchart of this study.

From January 2018 to August 2022, 936 patients diagnosed with first-ever ischemic stroke with an age between 18 and 80 years old were screened, 318 patients met at least one item of the exclusion criteria such as the presence of other central nervous disease, previous history of depression or anxiety, severe cognitive dysfunction or aphasia and were excluded, other 112 patients were excluded because of loss to or being unable to finish follow-up. At last, 506 patients met all the criteria and were enrolled in this study, 172 patients (33.93%) were identified to have PSD after 3 months, the other 334 patients were identified to have no PSD (details in Fig. 1).

The comparison between patients with and without PSD (Table1) showed no difference in the demographic characteristics, vascular risk factors and degree of WMH. However, there were some differences in lesion locations between two groups. Specifically, compared with patients without PSD, significantly more patients with PSD had infarctions in frontal lobe (P < 0.05), less of them had infarctions in brainstem (P < 0.05). The comparison of neuropsychological assessment showed significant difference between two groups. Patients of PSD had lower score of MMSE, BI and higher score of NIHSS and mRS (all P < 0.01).

**Table 1:** The characteristics of patients with and without PSD.

	PSD (n = 172)	Non-PSD (n = 334)	P
Demographic characteristics			
Male	50.6 (87)	52.4 (175)	0.708
Age	66.88 ± 8.01	67.53 ± 7.96	0.387
Education ≥ 6 years	59.3 (102)	61.7 (206)	0.631
Vascular risk factors			
Hypertension	75.0 (129)	71.9 (240)	0.462
Diabetes	41.9 (72)	36.5 (122)	0.248
Arrhythmia	11.6 (20)	11.7 (39)	0.999
Coronary artery disease	20.9 (36)	22.8 (76)	0.735
Smoking	30.2 (52)	29.0 (97)	0.837
Drinking	33.1 (57)	31.7 (106)	0.764
TOAST classification			
Large-artery atherosclerosis	26.7 (46)	24.6 (82)	0.591
Cardioembolism	26.2 (45)	18.6 (62)	**0.047**
Small-vessel occlusion	22.7 (39)	30.8 (103)	0.053
Others determined etiology	5.2 (9)	4.5 (15)	0.710
Undetermined etiology	19.2 (33)	21.6 (72)	0.533
Lesion location			
Frontal lobe	29.1 (50)	18.0 (60)	**0.006**
Parietal lobe	22.1 (38)	18.6 (62)	0.348
Temporal lobe	27.3 (47)	21.6 (72)	0.152
Occipital lobe	14.5 (25)	14.4 (48)	0.960
Basal ganglia	23.3 (40)	29.3 (98)	0.171
Brainstem	12.8 (22)	21.0 (70)	**0.028**
Cerebellum	18.6 (32)	21.0 (70)	0.561
WMH			
PWMH	1.26 ± 0.93	1.22 ± 0.99	0.660
DWMH	1.06 ± 0.86	1.09 ± 0.87	0.724
Neuropsychological assessment			
MMSE	24.54 ± 2.63	25.41 ± 2.70	**0.001**
NIHSS	5.92 ± 2.59	4.41 ± 2.49	**< 0.001**
BI	67.42 ± 11.56	73.76 ± 12.99	**< 0.001**
mRS	2.56 ± 1.29	1.92 ± 1.18	**< 0.001**
HAMD	3.79 ± 1.21	13.92 ± 5.11	**< 0.001**
Arrhythmia			
Previous history of arrhythmia	12.8 (22)	11.7 (39)	0.773
Post-stroke arrhythmia	51.2 (88)	37.1 (124)	**0.002**
Atrial fibrillation/flutter	19.8 (34)	14.7 (49)	0.163
Atrial tachycardia	16.9 (29)	9.0 (30)	**0.008**
Premature atrial contraction	20.9 (36)	16.8 (56)	0.274
Premature ventricular contraction	20.9 (36)	12.3 (41)	**0.008**
Ventricular tachycardia	6.4 (11)	3.3 (11)	0.112
Symptomatic arrhythmia	25.6 (44)	15.0 (50)	**0.05**
Newly-detected arrhythmia	39.5 (68)	24.3 (81)	**< 0.001**
Poor-response to AADs	23.8 (41)	13.2 (44)	**0.002**

Significant values are in bold.

The comparison about stroke subtypes (Table 1) showed that, patients of PSD had higher prevalence of cardioembolism (26.2% vs 18.6%, P < 0.05) than patient without PSD. Besides, the prevalence of small-vessel occlusion in PSD group seemed to be lower than that in non-PSD group (22.7% vs 30.8), however the comparison was not statistically significant (P = 0.053).

The comparison about various kind of arrhythmia (Table 1) showed that, there was no difference in the previous history of arrhythmia between two groups (P > 0.05). However, much more patients of PSD had post-stroke arrhythmia than those without PSD (P < 0.01). The detailed analysis about the subtypes of arrhythmia showed that, patients of PSD had higher prevalence of all kinds of ectopic arrhythmia including atrial arrhythmia/flutter, atrial tachycardia, premature ventricular/atrial contractions and ventricular tachycardia, although only the prevalence of atrial tachycardia and premature ventricular contractions were statistically different between two groups based on the sample size of this study (P < 0.01). Besides, more patients of PSD had newly-detected ectopic arrhythmia after stroke onset (P < 0.01), more patients of PSD had arrhythmia which was symptomatic and irresponsive to AAD after three months than those without PSD (both P < 0.01).

The results of logistic regression (Table 2) showed that, in all five different models, infarctions in frontal lobe, a lower score of MMSE and higher score of mRS were all independent risk factors for the presence of PSD after 3 months’ follow-up. Furthermore, previous history of arrhythmia and the presence of post-stroke arrhythmia regardless of previous history of arrhythmia were both not independently associated with the presence of PSD (P > 0.05), while newly-detected arrhythmia (OR 1.695, P < 0.05), symptomatic arrhythmia (OR 1.900, P < 0.05) and AAD-irresponsive arrhythmia (OR 2.002, P = 0.01) were all independently associated with the presence of PSD.

**Table 2:** Multivariate analysis for the independent predictor of PSD.

	OR	95% CI	P
Model1			
Frontal lobe	1.938	1.120–3.354	**0.018**
MMSE	0.854	0.787–0.926	**< 0.001**
NIHSS	1.116	1.001–1.244	**0.048**
mRS	1.248	1.024–1.522	**0.028**
Previous history of arrhythmia	1.028	0.548–1.928	0.931
Model2			
Frontal lobe	1.949	1.125–3.374	**0.017**
MMSE	0.853	0.787–0.925	**< 0.001**
NIHSS	1.109	0.995–1.236	0.061
mRS	1.238	1.014–1.510	**0.036**
Post-stroke arrhythmia	1.461	0.967–2.205	0.071
Model3			
Frontal lobe	1.896	1.095–3.285	**0.022**
MMSE	0.859	0.792–0.932	**< 0.001**
NIHSS	1.111	0.997–1.239	0.056
mRS	1.227	1.005–1.499	**0.044**
Newly-detected arrhythmia	1.695	1.099–2.614	**0.017**
Model4			
Frontal lobe	1.951	1.125–3.381	**0.017**
MMSE	0.851	0.784–0.923	**< 0.001**
NIHSS	1.111	0.996–1.239	0.058
mRS	1.256	1.028–1.534	**0.026**
Symptomatic arrhythmia	1.900	1.148–3.145	**0.013**
Model5			
Frontal lobe	2.093	1.202–3.643	**0.009**
MMSE	0.850	0.783–0.923	**< 0.001**
NIHSS	1.115	1.000–1.243	**0.050**
mRS	1.229	1.007–1.501	**0.043**
Poor-response to AAD	2.002	1.182–3.391	**0.010**

Significant values are in bold.

**Figure 2:**
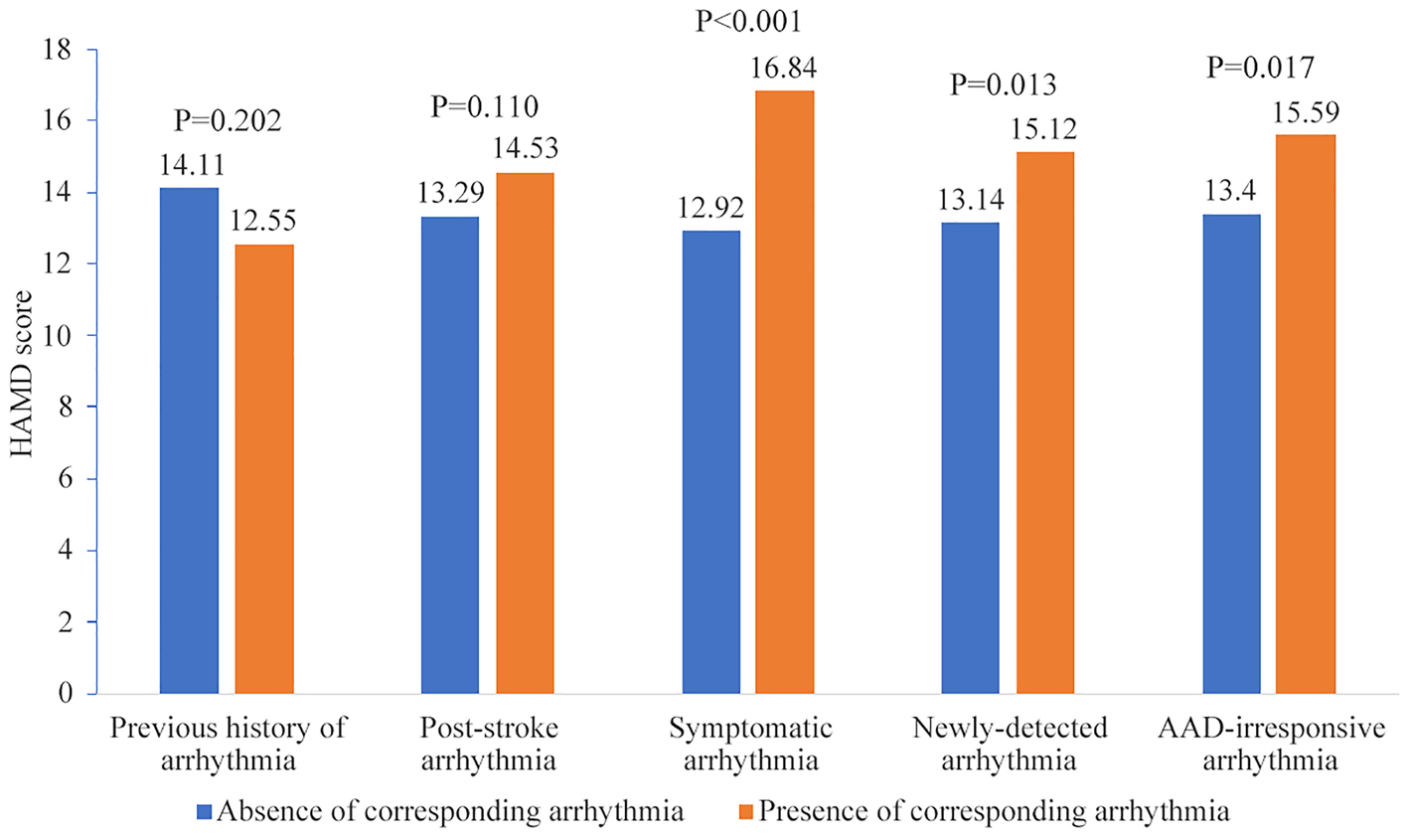
The impact of arrhythmia on the scores of HAMD among patients of PSD.

The comparison of HAMD score among patients with and without different kind of arrhythmia in PSD group (Table 3, Fig. 2) showed that, there was no difference of HAMD score either between patients with and without previous history of arrhythmia, or between patients with and without post-stroke arrhythmia (P > 0.05). However, patients with symptomatic arrhythmia had significantly higher score of HAMD than those without symptomatic arrhythmia in PSD group (16.84 ± 4.60 vs 12.92 ± 4.90, P < 0.01). Likewise, patients with newly-detected arrhythmia (15.12 ± 5.23 vs 13.14 ± 4.90, P < 0.05) and AAD-irresponsive arrhythmia (15.59 ± 5.09 vs 13.40 ± 5.02, P < 0.05) also had higher scores of HAMD than other patients in PSD group .

**Table 3:** The impact of arrhythmia on the scores of HAMD among patients of PSD.

	Absence of arrhythmia		Presence of arrhythmia		P
	Numbers	HAMD score	Numbers	HAMD score	
Previous history of arrhythmia	152	14.11 ± 5.12	20	12.55 ± 4.97	0.202
Post-stroke arrhythmia	84	13.29 ± 4.91	88	14.53 ± 5.25	0.110
Symptomatic arrhythmia	128	12.92 ± 4.90	44	16.84 ± 4.60	**< 0.001**
Newly-detected arrhythmia	104	13.14 ± 4.90	68	15.12 ± 5.23	**0.013**
AAD-irresponsive arrhythmia	131	13.40 ± 5.02	41	15.59 ± 5.09	**0.017**

Significant values are in bold.

## Discussion

This study showed that, post-stroke arrhythmia especially newly-detected arrhythmia after stroke and symptomatic arrhythmia were strongly associated with the presence of PSD and the severity of PSD after three months. Besides, the effective control of post-stroke arrhythmia was associated with reduced incidence and severity of PSD. As far as we know, this was the first report about the association between arrhythmia and PSD.

PSD has been a research focus about stroke for a long time, not only because of its high prevalence and adverse effect on the prognosis of stroke, but also because its pathogenesis which is complicated and puzzling to some extent. Previously various studies focused on the various neurological and psychological factors, such as lesion locations, severity of neurological deficits, handicap and cognitive dysfunction^9,20,21^. In this study, these factors were also analyzed. The results about lesion locations showed that patients with infarctions in the frontal lobe had a higher chance to develop PSD. This was consistent with a series of previous studies and was in favor of the positive correlation between frontal lobe and PSD^10,22,23^. The analysis about neurological deficits, handicap and cognitive dysfunction also showed positive results which was similar with previous reports^20,21^. However, partly due to the interference between neurological deficits and handicap, the association between PSD and each of them was weakened according to the results of logistic regression.

The main finding of this study was the association between arrhythmia and PSD. Arrhythmia was quite common in populations and was complicatedly associated with stroke. For example, atrial fibrillation was one of the major causes of ischemic stroke. Patients of stroke might have documented history of atrial fibrillation, or have atrial fibrillation detected after stroke^24^. Besides, other subtypes of ectopic arrhythmia were also quite common after stroke including atrial/ventricular tachycardia and frequent premature contraction. According to the results of this study, more than 1/3 patients had above-mentioned arrhythmia detected by long-term electrocardiogram after stroke. A large part of them had no history of ectopic arrhythmia. Although the percentage of previous ectopic arrhythmia was usually underestimated because some arrhythmia was symptomless and now known by patients, it is still proper to presume that a large part of newly-detected arrhythmia after stroke were triggered by stroke, i.e., a phenotype of stroke-heart syndrome. The detailed analysis about arrhythmia showed that, previous history of arrhythmia was not statically between patients with and without PSD, while post-stroke arrhythmia especially newly-detected arrhythmia was significantly associated with the occurrence of PSD. It suggested that newly-detected arrhythmia after stroke could be used as an early and distinguishable predictor of PSD.

The close association between post-stroke arrhythmia and PSD was probably based on their common pathogenesis. As a phenotype of stroke-heart syndrome, post-stroke arrhythmia was regarded as the result of brain–heart interaction with multiple pathogenesis involved just like PSD. Various clinical and experimental studies showed that sympathetic overdrive and reduced parasympathetic activity might be the key mediator between stroke and cardiac events including arrhythmia^25^. Besides, experimental studies showed that stroke-heart syndrome was accompanied by systemic inflammation and suggested that inflammation was involved in the pathogenesis of stroke-heart syndrome^26^. Similarly, autonomic dysfunction and inflammation were also important drivers of depression including post-stroke depression^27,28^. Studies about lesion locations also showed interesting results which suggested that post-stroke arrhythmia and PSD had common pathogenesis. For example, insula cortex which locates adjacent to frontal and temporal lobes is implicated in the process of complicated functions including autonomic control, emotions and empathy^29^. Various studies showed that infarctions in insula cortex were associated with post-stroke cardiac events especially arrhythmia^30–32^. Besides, injuries in amygdala, anterior cingulate cortex, ventromedial prefrontal cortex, hypothalamus, mediodorsal thalamus and hippocampus which were adjacent to insula cortex and supplied by middle cerebral artery might also lead to impairment of central autonomic networks^33,34^. Studies about the significance of lesion location in PSD showed quite controversial results. However, the most frequently-mentioned locations that might be associated with PSD were frontal and temporal lobes, amygdala and the fronto-limbic-striatal circuit^10,35,36^, which were also the locations involved in the adjustment of autonomic nervous system and post-stroke arrhythmia. Based on the similar pathogenesis with similar locations involved in their occurrence, it’s reasonable that the occurrence of post-stroke arrhythmia could predict the occurrence of PSD.

In this study, the results showed that post-stroke arrhythmia was associated with the severity of PSD. Besides, the management of post-stroke arrhythmia using AAD was associated with reduced incidence and severity of PSD. It suggested that post-stroke arrhythmia might not only indicate the presence of PSD-associated substrate such as the autonomic dysfunction and systemic inflammation, but also contributed to the occurrence of PSD via other mechanism. Previous studies have demonstrated that the presence of atrial and ventricular arrhythmia could lead to elevated level of psychological stress^16,37^, which was one major driver of depression^20^. It’s possible that post-stroke arrhythmia after stroke triggers continuous psychological distress after stroke and then leads to PSD. Psychological distress might also be the reason why patients with symptomless arrhythmia and AAD-responsive arrhythmia had lower scores of HAMD. Compared with symptomless arrhythmia, symptomatic arrhythmia might cause severer psychological distress^16^ and therefore lead to severer PSD. Successful control of post-stroke arrhythmia could alleviate psychological stress, then lead to a reduced incidence and severity of PSD.

However, based on the results of this study, it could be arbitrary to assert that arrhythmia leads to PSD. The results only suggested that post-stroke arrhythmia was associated with an elevated prevalence of PSD and might be a potential predictor of PSD; besides, successful management of arrhythmia was beneficial for the control of PSD. Actually, another direction about the association between arrhythmia and PSD should also be noticed. Although in this study, PSD was diagnosed three months after identical stroke, some patients might develop depression early after stroke^38^. As proven by previous studies, depression might also contribute to the development of arrhythmia via autonomic dysfunction or other mechanism^39,40^. It was possible that, the higher prevalence of post-stroke arrhythmia in PSD group might be partly contributed by early-onset depression.

## Conclusion

This study demonstrated that post-stroke arrhythmia especially newly-detected arrhythmia and symptomatic arrhythmia could be an early predictor of PSD. The common pathogenesis of post-stroke arrhythmia and PSD might be the main reason for the close association between them, with psychological distress being a possible mediator. Besides, successful management of arrhythmia could reduce the prevalence and severity of PSD.

### Limitation

First, the presence of previous history of arrhythmia was based on medical record and information supplied by patients, and could be underestimated. Second, the presence of post-stroke arrhythmia was based on the results of 3-day continuous electrocardiogram or monitor and could also be underestimated because some patients might only have episodes of arrhythmia after 3 days, and also be underestimated for soma patients who didn’t carry a monitor for a longer time. Third, part of patients with atrial fibrillation were excluded because of severe neurological deficits and made the selective bias inevitable. Anyway, in the future, more relevant studies about the predictor of PSD and the association between post-stroke arrhythmia and PSD were still required.

## Data Availability

The raw data supporting the conclusions of this article will be made available by the authors. If someone wants to request the data from this study, please contact Dr. Tao Xu, Email: 22118211@zju.edu.cn.

## Abbreviations

PSD: Post-stroke depression
AAD: Anti-arrhythmic drugs
MMSE: Mini-Mental State Examination
NIHSS: National Institutes of Health Stroke Scale
HAMD: Hamilton Depression Scale
DSM-IV: Diagnostic and Statistical manual of mental disorders, 4th edn
BI: Barthel Index
mRS: Modified Rankin Scale
WMH: White matter hyperintensities
